# Multi-dimensional analysis of adult acute myeloid leukemia cross-continents reveals age-associated trends in mutational landscape and treatment outcomes (Acute Myeloid Leukemia Cooperative Group & Alliance for Clinical Trials in Oncology)

**DOI:** 10.1038/s41375-025-02644-0

**Published:** 2025-09-19

**Authors:** Monica Cusan, Karilyn Larkin, Deedra Nicolet, Vindi Jurinovic, Krzysztof Mrózek, Aarif M. N. Batcha, Maja Rothenberg-Thurley, Stephanie Schneider, Cristina Sauerland, Dennis Görlich, Utz Krug, Wolfgang E. Berdel, Bernhard J. Woermann, Wolfgang Hiddemann, Jan Braess, Karsten Spiekermann, Philipp A. Greif, James S. Blachly, Alice S. Mims, Christopher J. Walker, Michael C. Walker, Christopher C. Oakes, Shelley Orwick, Andrew J. Carroll, William G. Blum, Bayard L. Powell, Jonathan E. Kolitz, Joseph O. Moore, Robert J. Mayer, Richard A. Larson, Richard M. Stone, John C. Byrd, Klaus H. Metzeler, Tobias Herold, Ann-Kathrin Eisfeld

**Affiliations:** 1 Laboratory for Leukemia Diagnostics, Department of Medicine III, University Hospital, LMU Munich, Munich, Germany; 2 Division of Hematology, Department of Internal Medicine, The Ohio State University Comprehensive Cancer Center, Columbus, OH USA; 3 The Clara D. Bloomfield Center for Leukemia Outcomes Research, The Ohio State University Comprehensive Cancer Center, Columbus, OH USA; 4 Alliance Statistics and Data Management Center, The Ohio State University Comprehensive Cancer Center, Columbus, OH USA; 5 Institute for Medical Information Processing, Biometry and Epidemiology, LMU Munich, Munich, Germany; 6 DIFUTURE (Data integration for Future Medicine), LMU Munich, Munich, Germany; 7 Institute of Human Genetics, University Hospital, LMU Munich, Munich, Germany; 8 Institute of Biostatistics and Clinical Research, University of Münster, Münster, Germany; 9 Department of Medicine 3, Klinikum Leverkusen, Leverkusen, Germany; 10 Department of Medicine, Hematology and Oncology, University of Münster, Münster, Germany; 11 German Society of Hematology and Oncology, Berlin, Germany; 12 German Cancer Consortium (DKTK), Partner Site Munich, Munich, Germany; 13 German Cancer Research Center (DKFZ), Heidelberg, Germany; 14 Department of Oncology and Hematology, Hospital Barmherzige Brüder, Regensburg, Germany; 15 Department of Genetics, University of Alabama at Birmingham, Birmingham, AL USA; 16 Emory University School of Medicine, Atlanta, GA USA; 17 Wake Forest Baptist Comprehensive Cancer Center, Winston-Salem, NC USA; 18 Zuckerberg Cancer Center, Hofstra Northwell School of Medicine, Lake Success, NY USA; 19 Duke Cancer Institute, Duke University, Durham, NC USA; 20 Dana-Farber Cancer Institute, Boston, USA; 21 University of Chicago, Chicago, IL USA; 22 Department of Medical Oncology, Dana-Farber/Partners CancerCare, Boston, MA USA; 23 Department of Internal Medicine, University of Cincinnati, Cincinnati, OH USA; 24 Department of Hematology, Cellular Therapy, Hemostaseology and Infectious Diseases, Leipzig University Hospital, Leipzig, Germany

**Keywords:** Acute myeloid leukaemia, Cancer genetics, Prognosis

## Abstract

The outcome of patients with acute myeloid leukemia (AML) worsens with increasing age. Dichotomization into “younger” and “older” patients is clinically routine and often dictates treatment options. We aimed to delineate whether molecular genetic features and/or outcome measures support assorting patient populations by age, including division into “younger” and “older” groups. We analyzed 2823 adult AML patients enrolled onto frontline chemotherapy-based clinical protocols of two cooperative study groups from USA and Germany who were profiled molecularly via targeted sequencing platforms. Frequencies of gene mutations and cytogenetic findings were depicted in 5-year age increments. Clinical outcomes of 2756 AML patients were analyzed with respect to molecular features, genetic-risk groups and age. Age-associated distributions of gene mutations and cytogenetic abnormalities were similar in both cohorts. There was almost linear shortening of overall survival with increasing age among all patients (*P* < 0.001) and within 2022 European LeukemiaNet-defined genetic-risk groups, with survival decreasing as age increased (favorable-risk, *P* < 0.001; intermediate-risk, *P* < 0.001; adverse-risk, *P* < 0.001). Although mutational profiles and outcomes of the youngest patients differed from those of older patients, there was no age cut-off identifying “younger” and “older” patients. These findings support more age-associated flexibility for drug approval and trial eligibility.

## Introduction

Acute myeloid leukemia (AML) is a disease affecting predominantly older patients, but it does occur across the entire age spectrum. Patient outcomes strongly correlate with patient-associated factors such as age, race and performance status as well as disease-associated factors such as AML-associated cytogenetic [1–3] and molecular genetic abnormalities [4–7]. The contribution of increasing age to worsening survival has been well established [8, 9] and, as a consequence, age currently represents a major factor in the consideration of treatment options [10], including curative intensive chemotherapy regimens, eligibility for many clinical trials and has even found a place on the United States (US) Food and Drug Administration (FDA) label for one targeted therapy [11]. Notably, this aged-based distinction derives from the observation that AML in the elderly is a distinct clinical entity compared with the AML in younger patients, as well as the assumption of concurrent decreasing fitness and increasing comorbidities, an assumption that has rightly been challenged with the rise of more comprehensive and objective geriatric assessments [4, 10, 12–15].

However, our understanding of the underlying disease biology and driver mutations has tremendously improved and at last translated into both novel targeted therapies and effective combinations outside of intensive induction regimens [16–19]. Thus, in the current era of expedient genomic classification [20–22], the question of relevance of age alone in AML - rather than the presence or absence of targetable molecular features or genetic risk groups - is again being challenged [5, 6, 23–32]. Age as a major criterion for inclusion (or exclusion) from clinical trials could preclude patients from getting an effective therapy, and could bias and impede our understanding of the biology of disease as well as responses to therapy [33]. Distinct age cut-offs between the different study cohorts can confound interpretation of the results and could result in loss of valuable information.

In the present work, we performed a multi-dimensional analysis of mutational patterns and survival correlations through 5-year age intervals from 18 to 92 years, using two large datasets of de novo AML patients from Germany and USA. The aims of this study were: 1) to perform an unbiased characterization of the molecular landscape across the age spectrum of adult AML, 2) to analyze the survival of adult AML patients receiving similar, frontline chemotherapy on clinical trials stratified by age groups, and 3) to evaluate the rationale for age cuts used in the characterization and treatment guidance of AML via integration of molecular, clinical and survival parameters when traditional chemotherapy is utilized.

## Methods

### Patients and treatment

**Fig. 1:**
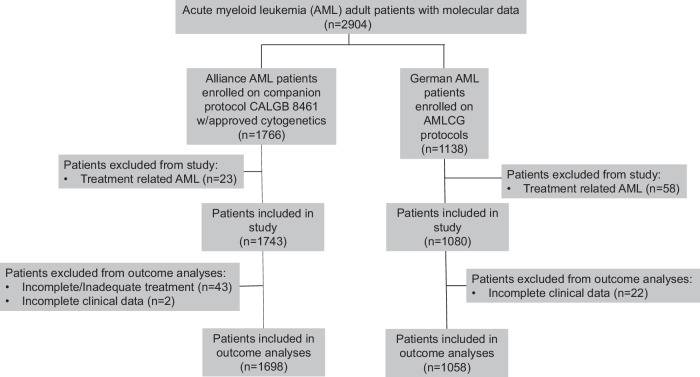
A CONSORT diagram indicating the inclusion and exclusion criteria for the patients data analyzed in the present study. CALGB Cancer and Leukemia Group B, AMLCG AML Cooperative Group.

Our combined patient cohort comprised 2823 patients diagnosed with AML (other than acute promyelocytic leukemia) who were treated in the setting of frontline treatment protocols of two large cooperative groups, including 1743 patients from the Cancer and Leukemia Group B (CALGB) enrolled between 1986 and 2016 [34–48], and 1080 patients enrolled on protocols of the AML Cooperative Group (AMLCG) between 1999 and 2017 [8, 49, 50] (for details see Supplementary Information). CALGB is now part of Alliance for Clinical Trials in Oncology (Alliance). Treatment of all patients included in outcome analyses included intensive cytarabine-based induction therapy. Performance of allogeneic hematopoietic stem-cell transplantation (HSCT), on or off protocol, was considered as independent variable. The treatment regimens are described in the Supplementary Information. The CONSORT diagram with the patient inclusion/exclusion criteria in this study is shown in Fig. 1. For subsequent outcome analyses, patients who received incomplete or inadequate treatment were excluded, resulting in a total of 2756 patients included in the outcome analyses (CALGB/Alliance, *n *= 1698; AMLCG, *n *= 1058; Fig. 1).

### Cytogenetic and molecular analyses

Cytogenetic analyses of pretreatment bone marrow and/or blood samples subjected to short-term (24- or 48-h) unstimulated cultures were performed by the CALGB/Alliance- and AMLCG-approved institutional laboratories, and the results were confirmed by central karyotype review [51] (Supplementary Information).

Patients in both cohorts were profiled for molecular features via targeted sequencing platforms. The respective US and German targeted molecular panels contained 24 shared AML-associated genes. We used a variant allele frequency (VAF) cut-off ≥2%, which represents the lower limit of detection for most clinically used next generation sequencing assays, and as previously reported by our groups [5, 27]. In the analysis of mutation frequency in the entire cohort, we focused on those mutations that occurred in at least 4% of patients [5, 27, 52]. Frequency determination of selected gene mutations and cytogenetic findings in both datasets was done in age groups, first comprising patients aged 18–24 years and then by 5-year intervals until the age of 74 years and finally for patients aged 75 years or older (range, 75–92).

### Statistical analyses

Definitions of clinical endpoints are provided in the Supplementary Information. Early death (ED) is defined as death within 30 days after protocol enrollment. Estimated probabilities of OS were calculated using the Kaplan-Meier method. Hazard ratios (including 95% confidence intervals [CIs]) were estimated from Cox proportional hazard models. Analyses were performed by the Alliance Statistics and Data Management Center on a database locked on February 11, 2021, using SAS 9·4, TIBCO Spotfire S + 8·2 and GraphPad Prism version 10.

## Results

### Clinical characteristics of AML patients

The median age was 55 years (range, 18–92 years), with 45% of patients being female. Thirty-four percent of patients belonged to the 2022 European LeukemiaNet (ELN) favorable genetic-risk group, and 27% and 39% were classified as having intermediate or adverse genetic risk, respectively. Seventy-nine percent of patients were either fully active or ambulatory (ECOG 0-1) with respect to their performance status at time of diagnosis. Twenty-six percent of patients received an allogeneic HSCT in first CR (Table 1). Pretreatment characteristics of patients in the US and German cohorts are provided in Supplementary Table 1. The median follow-up for patients who are alive is 8.2 years.

**Table 1:** Pretreatment characteristics and outcomes of patients with AML included in our study.

Characteristic	All patients *n* = 2823
Age, years	
Median	55
Range	18–92
Sex, no. (%)	
Female	1270 (45)
Male	1553 (55)
Hemoglobin, g/dL	
Median	9.1
Range	2.3–25.1
Platelet count, ×10^9^/L	
Median	55
Range	0–1760
WBC count, ×10^9^/L	
Median	22.2
Range	0.1–798.2
Bone marrow blasts, %	
Median	70
Range	0-100
Performance status, no. (%)	
Fully active	654 (30)
Ambulatory	1085 (49)
In bed <50% of the time	356 (16)
In bed >50% of the time	88 (4)
Completely bedridden	16 (1)
2022 ELN, no. (%)	
Favorable	944 (35)
Intermediate	721 (27)
Adverse	1067 (39)
Treatment in first CR, no. (%)	
Chemotherapy	1329/1807 (74)
Allogeneic HSCT	478/1807 (26)
CR rates, no. (%)	
Achieved CR	1850 (66)
Did not achieve a CR	973 (34)
Overall survival, % (95% CI)	
Alive at 3 years	35 (33–37)

*WBC* white blood cell, *ELN* European LeukemiaNet, *CR* complete remission, *HSCT* hematopoietic stem cell transplantation.

### AML-related gene mutations follow different patterns over time

**Fig. 2:**
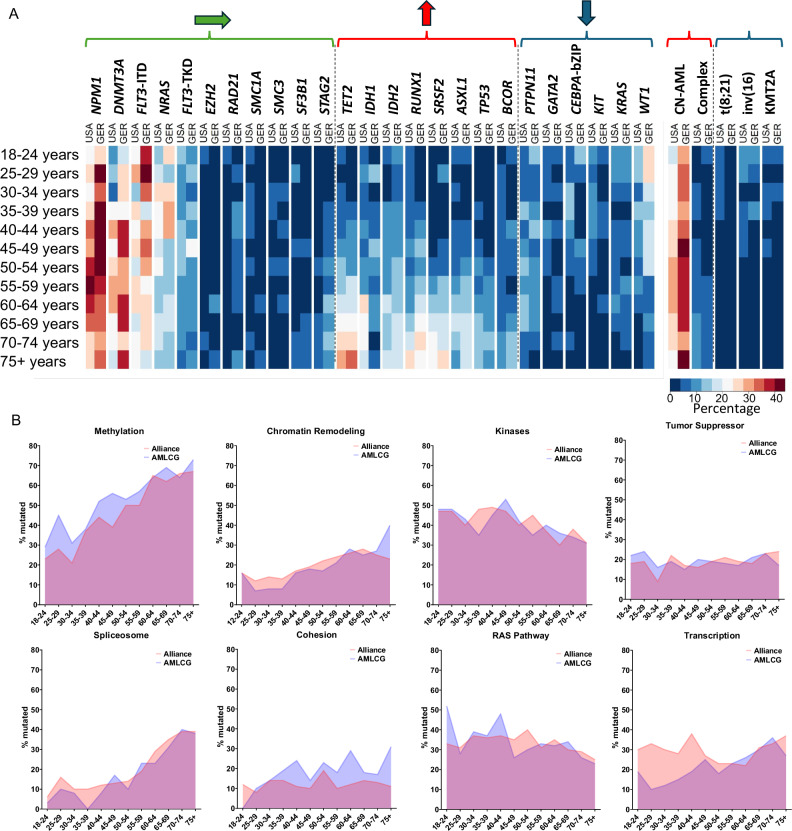
Mutational analysis of patients with AML in the US and German cohorts. **A** Heatmap showing the frequency of the common mutations in 5-year age intervals. Three groups with different patterns of age-related occurrence of genetic alterations are indicated by color: green, mutations with non-linear age-frequency distribution; red, genetic alterations whose frequency increased with increased age; and blue, alterations whose frequency decreased with increased age. The arrows indicate direction of changes with age for these three patterns. **B** Area plots showing the frequencies of mutations grouped by their biological category (as previously described by Cancer Genome Atlas Research Network [4]) in age intervals.

We analyzed the genetic alterations across the age spectrum of adult patients with AML (from 18 to 75+ years) and found age-associated frequency patterns of gene mutations and recurrent cytogenetic abnormalities. The *first* group comprised alterations with non-linear age-frequency distribution, which included three most common AML-associated gene mutations, that is, mutations in the *NPM1* and *DNMT3A* genes and *FLT3* internal tandem duplication (*FLT3*-ITD), *FLT3* tyrosine kinase domain mutations (*FLT3*-TKD), *NRAS* and *EZH2* mutations and mutations affecting the cohesin complex genes (*RAD21*, *SMC1A*, *SMC3*, *SF3B1*, *STAG2*). The *second* group consisted of genetic alterations whose frequency increased with age, namely mutations in the *ASXL1*, *BCOR*, *IDH1, IDH2*, *RUNX1*, *SRSF2*, *TET2* and *TP53* genes and both complex and normal karyotypes. The *third* group included alterations whose frequency decreased with increased age, such as mutations in the *CEBPA*, *GATA2*, *KIT*, *KRAS*, *PTPN11* and *WT1* genes, and core-binding factor balanced rearrangements [i.e., t(8;21)(q22;q22) and inv(16)(p13.1q22)/t(16;16)(p13.1;q22)] and rearrangements involving band 11q23 and the *KMT2A* (formerly *MLL*) gene (Fig. 2A, Supplementary Tables 2–4). These patterns were strikingly similar between the US and German cohorts (Fig. 2A). Notably, we could not identify a distinct age cut-off that could delineate a specific age at which the patterns of genetic alterations would noticeably change consistently across different mutations. This was also true for previously defined functional groups [4] (Fig. 2B, Supplementary Table 5).

### OS diminishes with increasing age in adult patients with AML

**Fig. 3:**
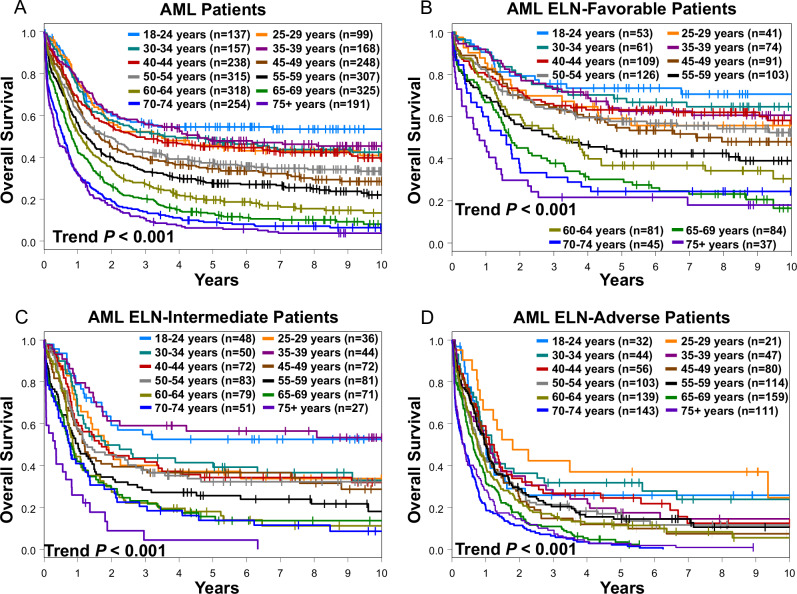
Overall survival (OS) of patients from both cohorts divided by age intervals. **A** OS of all patients. **B**–**D** OS of patients assigned to the 2022 ELN genetic-risk groups: **B** favorable, **C** intermediate and **D** adverse.

The 5-year OS rate of all patients in our study was 40%, and there was a positive correlation between shortening of OS and increasing age (Fig. 3A). This age-associated worsening of OS was consistent across 2022 ELN genetic-risk groups. In the 2022 ELN favorable genetic-risk patients, the estimated proportion of patients alive at 5 years ranged from 21% for those aged 75 years or older to 73% for the youngest patients (18–24 years old; Fig. 3B). Similarly, 2022 ELN intermediate genetic-risk patients followed this survival pattern, with 5-year OS rate of 4% in patients aged ≥75 years compared with 53% in those aged 18–24 years (Fig. 3C). In 2022 ELN adverse genetic-risk patients, we found, expectedly, less variability, with the lowest 5-year OS rate of 2% in patients aged 70–74 years compared with the highest 5-year OS rate of 37% in patients aged 25–29 years (Fig. 3D). Thus, although application of the 2022 ELN criteria allows risk stratification of patients with AML, age itself is also an important factor with regard to determining OS within each 2022 ELN genetic-risk group. Consistent with the aforementioned results, we found no specific age that would serve as a cut-off point to identify patients with better and those with worse OS within each of the 2022 ELN genetic-risk group, further supporting age as a continuum in AML for both biology and risk stratification.

Concerning the CR and ED rates in age intervals, we observed a trend in CR rates decreasing with age and a trend in ED rates increasing with age, although again without a clear age-dependent delineator (Table 2). Of note, all results mentioned above were comparable in the US and German cohorts when each cohort was considered separately (data not shown).

**Table 2:** Summary of complete remission and early death rates per age groups.

Outcome endpoint	Age group											
	18–24 years (*n* = 137)	25–29 years (*n* = 99)	30–34 years (*n* = 157)	35–39 years (*n* = 168)	40–44 years (*n* = 238)	45–49 years (*n* = 248)	50–54 years (*n* = 315)	55–59 years (*n* = 307)	60–64 years (*n* = 318)	65–69 years (*n* = 325)	70–74 years (*n* = 254)	75 + years (*n* = 190)
CR rate	78%	79%	75%	82%	78%	70%	72%	65%	64%	57%	47%	41%
ED rate	3%	2%	1%	2%	5%	6%	7%	8%	7%	14%	19%	24%

*CR* complete remission, *ED* early death.

### Age-dependent outcome varies along mutational subgroups

We then assessed the impact of age on outcome of select groups of patients harboring specific, recurring, AML-associated gene mutations, with focus on mutations for which approved targeted inhibitors exist, including mutations in the *IDH1* and *IDH2* genes, *FLT3-*ITD and *FLT3-*TKD. Of note, for these targeted inhibitors, distinct age cut-offs were introduced in the trials testing them and thus led to questions about the generalizability of results in subsequent clinical practice [7, 41, 53].

**Fig. 4:**
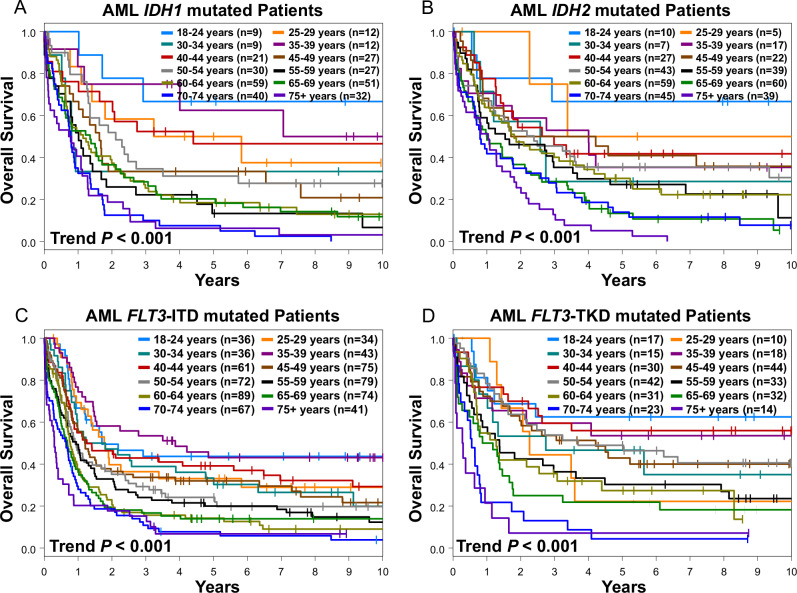
Overall survival of patients with AML harboring clinically relevant mutations who were grouped by age intervals. **A** Patients with *IDH1* mutations, **B** *IDH2* mutations, **C** *FLT3*-ITD, and **D** *FLT3*-TKD.

**Fig. 5:**
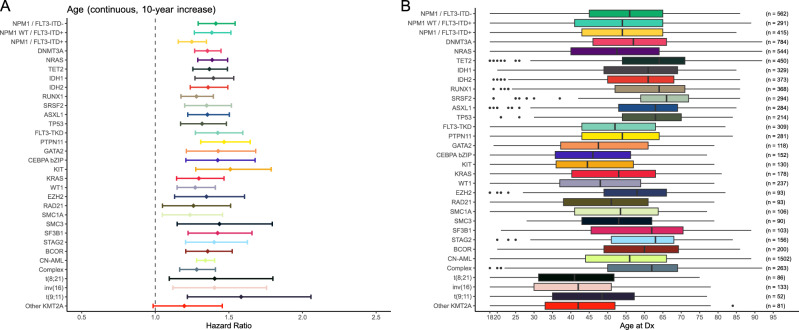
Relevance of age on patient outcome and the distribution of age of diagnosis for individual mutations. **A** Forrest plot showing the hazard ratio for survival per mutation by age. **B** Plot demonstrating the age of diagnosis distribution for each mutation.

In these molecularly defined subgroups, we examined patient survival and found that age in fact has a negative prognostic impact within the *IDH1* and *IDH2* mutations groups and in both *FLT3*-ITD and *FLT3*-TKD (Fig. 4A–D). To further investigate the relevance of age on outcome for individual mutations, we calculated the hazard ratio of age in the mutational subgroups, considering age as a continuous variable. In this analysis, increasing age associated with inferior survival in a significant way in all prognostically relevant AML-associated gene mutations and chromosome rearrangements we analyzed, with the exception of rearrangements involving 11q23/*KMT2A* other than t(9;11)(p22;q23) (Fig. 5). The median age of patients with each mutation did not correlate with the magnitude of the effect that age had on risk of death, which is to say that within each individual mutation grouping, it did not matter whether a mutation was more common in younger or older patients; the spectrum of age remains an important factor in each case. In conclusion, association of age with prognosis can only in part be attributed to certain gene distributions and the reasons for age-dependent prognosis within certain gene groups remain elusive.

## Discussion

To our knowledge, this is one of the first large scale cross-continent depictions of mutational patterns and outcome in AML inclusive of the entire adult age spectrum. We characterize different age-associated molecular distribution patterns that do not provide support for a molecular basis of a singular age that would justify a younger versus older age categorization. Importantly, it was not our scientific objective to define precise cut points among age groups, but instead we aimed to depict molecular patterns across ages, and test whether associated survival patterns may outweigh any age-restricted definitions.

While this study sought to challenge the relevance of chronologic age in AML, we have in fact shown that age provides further risk stratification to patients already classified by 2022 ELN in an almost continuous fashion, with substantive differences in OS between the youngest and oldest patients belonging to the same 2022 ELN genetic-risk group. The age-associated survival was less pronounced in the adverse risk group, which is not surprising given that overall outcomes of patients in this group are poor, suggesting that the weight of adverse risk disease features supersedes the importance of age-related factors in this risk group.

When we examined the prognostic impact of prognostically relevant AML-associated gene mutations and recurrent chromosome abnormalities with respect to age, we found that the established negative survival association of most of these alterations worsened with increasing age (Fig. 5). Similarly, some genetic abnormalities associated with favorable outcome such as *NPM1* mutations without *FLT3*-ITD and inv(16) also tended to lose their favorable influence as patients aged. In contrast, the poor prognostic impact of 11q23/*KMT2A* rearrangements other than t(9;11) was independent of age; but will require additional validation due to the relatively small sample size. Surprisingly, this was not the case for other known adverse risk subtypes, such as *TP53*, complex karyotype or *FLT3*-ITD mutation groups, although they had relatively lower hazard ratios. This provides a rationale for individualized risk- and associated treatment-eligibility assessments that take both age and molecular features into account.

This study is limited to patients who met eligibility requirements for clinical trials, which means that subsets of real-world patients with more severe organ dysfunction, uncontrolled infections and concurrent malignancies are not included in our study. Additionally, owing to a relatively small sample size of Hispanic or Black patients, caution should be applied when considering how our findings might relate to these patient groups, and underscores the importance of diversity and inclusivity considerations for all future trial design and enrollment. Similarly, while an analysis of the contribution of allogeneic stem cell transplant to outcomes by age and ELN risk group would be ideal, lack of complete data for all patients (concurrent performance status, co-morbidity index) and differences between US and German application of transplant in consolidation preclude an unbiased look at the specific impact of transplant, and this needs to be carefully evaluated in future work.

In conclusion, choosing a precise age cut-off such as age 39 years for separating adolescents and young adult patients from older adults, or 59 years for identifying “younger” and “older” AML patients, does not seem to be supported by the results of our analyses. While our intention was to assess whether defined age cut-offs are supported by patterns of genetic alterations, our data tend to refute the existence of any uniform cut-offs across the age spectrum, whether considering the younger adults population aged 18–39 years, or the distinction between “younger and older” AML patients with age greater than 55 or 60 years defining older patients with AML.

Previous reports have already described age-associated differences in mutation frequencies [5, 6, 52, 54]. However, these observations have been limited by the comparison between only two pre-defined age groups, such as <60 versus as ≥60 years, or children/adolescents versus adults. In our study, we did analyze age using 5-year intervals, and this allowed us to observe different age patterns for different mutations. The shortcomings associated with the use of age as a dichotomized variable for therapy decisions or inclusion in clinical trials are also supported by several reports that revealed strong age disparities between clinical trials and “real” world data [33, 55, 56]. This is exemplified by 18% of AML patients <60 years harboring either *IDH1* or *IDH2* mutations, which would qualify them for the use of a targeted inhibitor but who might be excluded from receiving this therapy given their younger age.

The last few years have seen the exciting translation of biologic drivers in specific subsets of AML into targets of inhibitors that have demonstrated single agent activity in relapsed and refractory disease, as well as increased survival when used in combination with standard therapies [41, 57]. It is hoped that in the future, the number of patients who are treated with these targeted therapies and have a longer follow-up time will increase sufficiently to enable performing studies similar to the one we report here, including analyses of age influence on patient outcomes within the 2022 ELN genetic-risk groups and among patients harboring specific, prognostically relevant driver mutations and/or cytogenetic abnormalities. This will allow determination of whether our conclusions hold-up in patient populations treated differently from the one we studied, that is patients receiving intensive chemotherapy only. Moreover, integrated algorithms based on age, mutational profiles and performance status will likely be developed that would aid in making individual therapy decisions. Together with increased openness adopted by all clinical trials, this will give the chance to all patients to get the best available therapy tailored to their individual characteristics.

## Supplementary information

Supplemental Table 1

Supplemental Tables 2-5

Supplemental Materials

## Data Availability

Patient data used in survival analyses were obtained from the Alliance Statistics and Data Management Center and the AMLCG database. Individual participant data will not be shared. Legal restrictions prohibit us from publicly sharing raw sequencing data, which, however, could be made available upon reasonable request and permission of the local ethics committee.
