# Supplementary material for: Multi-dimensional analysis of adult acute myeloid leukemia cross-continents reveals age-associated trends in mutational landscape and treatment outcomes (Acute Myeloid Leukemia Cooperative Group & Alliance for Clinical Trials in Oncology)
Source: Leukemia. 2025 Sep 19;39(12):2926–34. doi: 10.1038/s41375-025-02644-0 (PMC12634432; doi:10.1038/s41375-025-02644-0)
Supplement: Supplementary file 1 — Supplemental Table 1 [file 41375_2025_2644_MOESM1_ESM.docx]

**Supplementary Table 1.** Pretreatment characteristics and outcomes of the US and German patients with AML included in our study

| **Characteristic** | **US patients**  **(n=1743)** | **German patients**  **(n=1080)** |
| --- | --- | --- |
| Age, years |  |  |
| Median   Range | 53  18-92 | 58  18-86 |
| Sex, no. (%) |  |  |
| Female | 754 (43) | 516 (48) |
| Male | 989 (57) | 564 (52) |
| Hemoglobin, g/dL |  |  |
| Median   Range | 9.2  2.3-25.1 | 9.0 3.5-16.0 |
| Platelet count, x10^9^/L |  |  |
| Median   Range | 55  4-989 | 55  0-1760 |
| WBC count, x10^9^/L |  |  |
| Median   Range | 23.5  0.4-560.0 | 20.4  0.1-798.2 |
| Bone marrow blasts, % |  |  |
| Median   Range | 66  0-99 | 80  6-100 |
| Performance status, no. (%) |  |  |
| Fully active | 464 (31) | 190 (27) |
| Ambulatory | 746 (50) | 339 (48) |
| In bed <50% of the time | 222 (15) | 134 (19) |
| In bed >50% of the time | 51 (3) | 37 (5) |
| Completely bedridden | 10 (1) | 6 (1) |
| 2022 ELN, no. (%) |  |  |
| Favorable | 589 (36) | 355 (33) |
| Intermediate | 432 (26) | 289 (27) |
| Adverse | 634 (38) | 433 (40) |
| Treatment in first CR, no. (%) |  |  |
| Chemotherapy | 1377 (82) | 749 (69) |
| Allogeneic HSCT | 300 (18) | 331 (31) |
| CR rates, no. (%) |  |  |
| Achieved CR | 1121 (64) | 729 (68) |
| Did not achieve a CR | 622 (36) | 351 (33) |
| Overall survival, % (95% CI) |  |  |
| Alive at 3 years | 32 (30-34) | 39 (36-42) |

Abbreviations: WBC, white blood cell counts; ELN: European LeukemiaNet; CR, complete remission; HSCT, hematopoietic stem cell transplantation.
