# Supplementary figures and images for: Multi-dimensional analysis of adult acute myeloid leukemia cross-continents reveals age-associated trends in mutational landscape and treatment outcomes (Acute Myeloid Leukemia Cooperative Group & Alliance for Clinical Trials in Oncology)
Source: Leukemia. 2025 Sep 19;39(12):2926–34. doi: 10.1038/s41375-025-02644-0 (PMC12634432; doi:10.1038/s41375-025-02644-0)

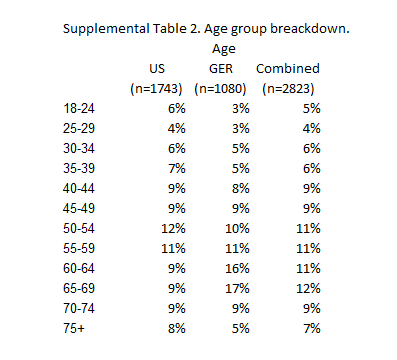


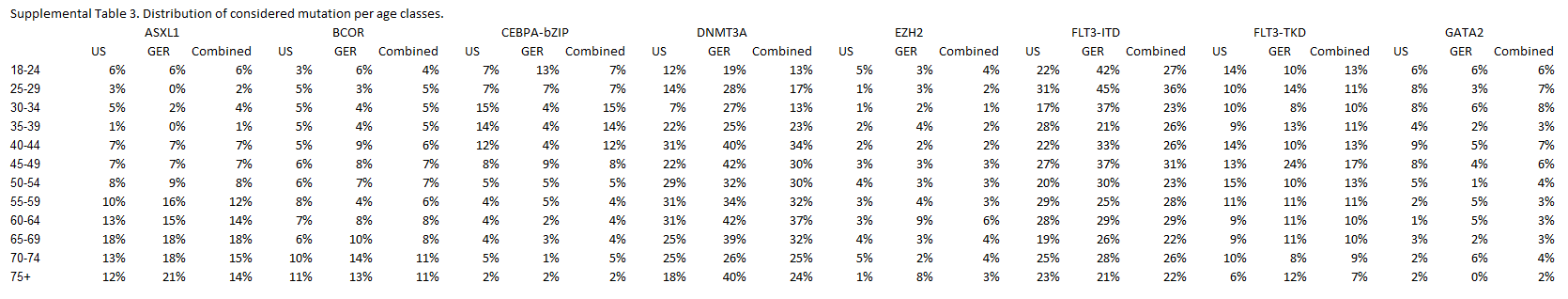


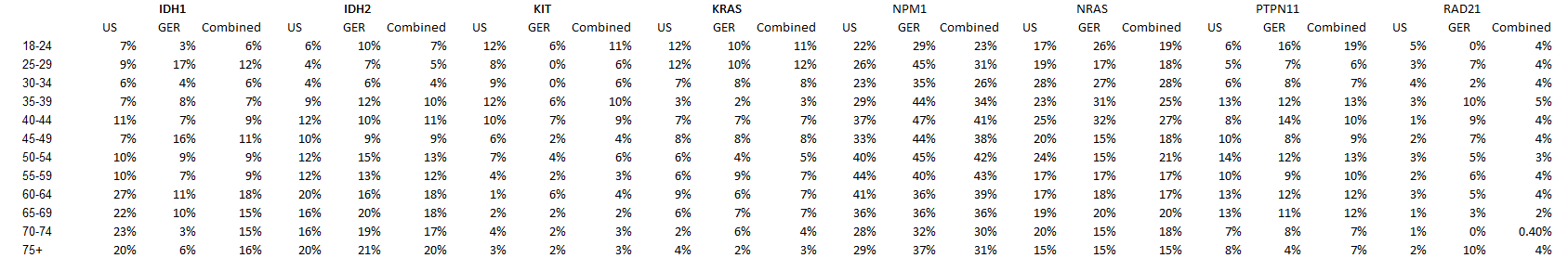


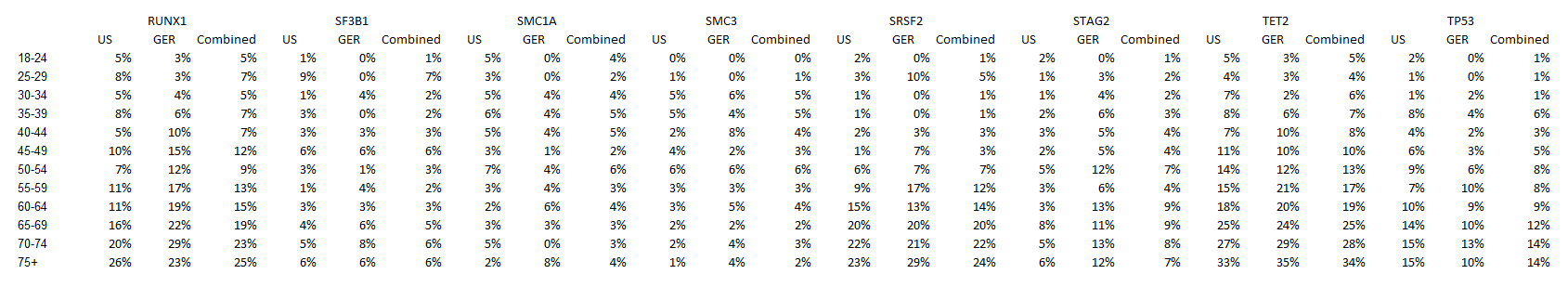


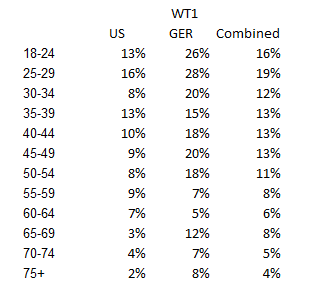


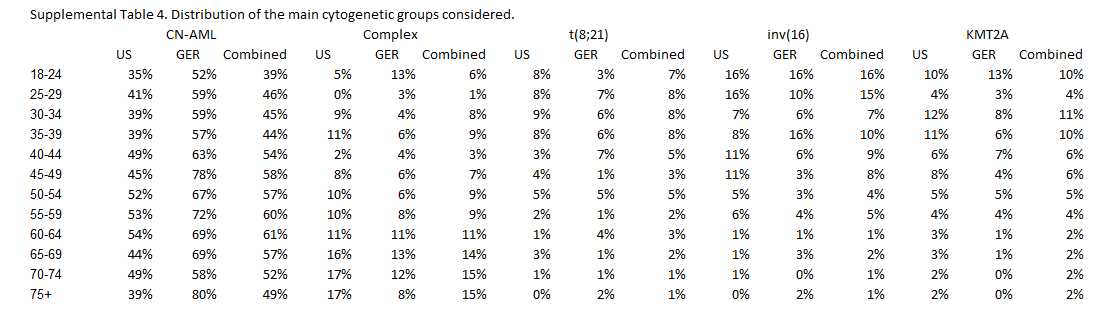


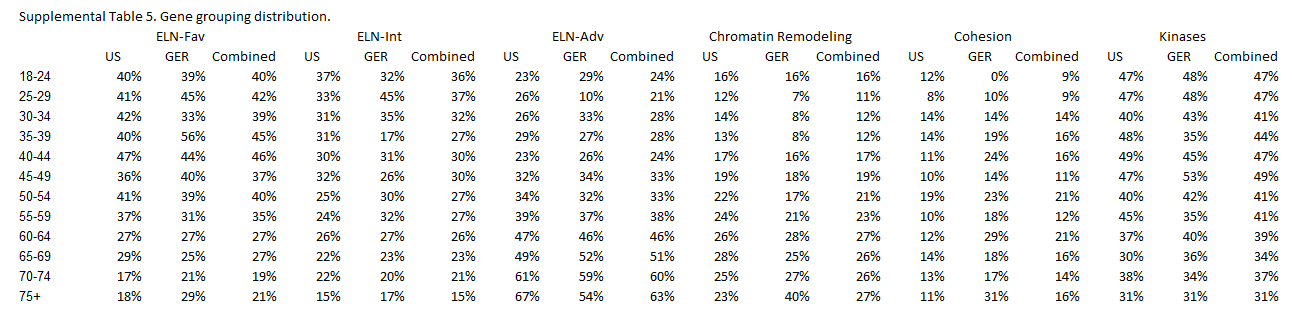


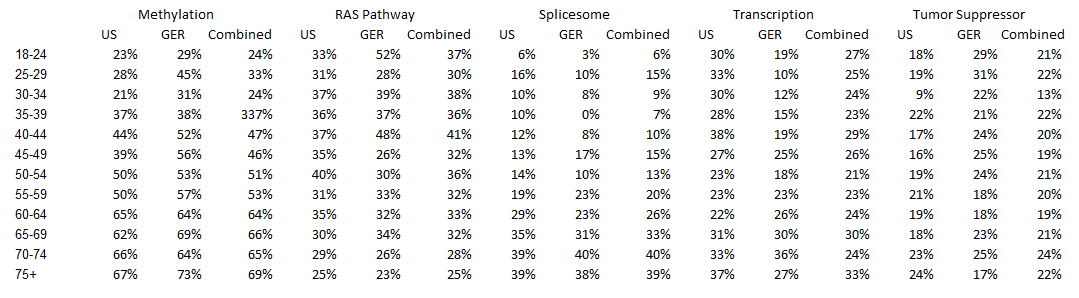

Supplement: Supplementary file 2 — Supplemental Tables 2-5 [file 41375_2025_2644_MOESM2_ESM.docx]
