# Supplementary material for: Multi-dimensional analysis of adult acute myeloid leukemia cross-continents reveals age-associated trends in mutational landscape and treatment outcomes (Acute Myeloid Leukemia Cooperative Group & Alliance for Clinical Trials in Oncology)
Source: Leukemia. 2025 Sep 19;39(12):2926–34. doi: 10.1038/s41375-025-02644-0 (PMC12634432; doi:10.1038/s41375-025-02644-0)
Supplement: Supplementary file 3 — Supplemental Materials [file 41375_2025_2644_MOESM3_ESM.docx]

**Supplementary Information**

**Multi-dimensional analysis of adult acute myeloid leukemia cross-continents reveals age-associated trends in mutational landscape and treatment outcomes (AMLCG & Alliance)**

Monica Cusan,^1,^* Karilyn Larkin,^2,3,^* Deedra Nicolet,^3,4^ Vindi Jurinovic,^1^

Krzysztof Mrózek,^3^ Aarif M. N. Batcha,^5,6^ Maja Rothenberg-Thurley,^1^

Stephanie Schneider,^1,7^ Cristina Sauerland,^8^ Dennis Görlich,^8^ Utz Krug,^9^

Wolfgang E. Berdel,^10^ Bernhard J. Woermann,^11^ Wolfgang Hiddemann,^1,12,13^

Jan Braess,^14^ Karsten Spiekermann,^1,12,13^ Philipp A. Greif,^1,12,13^ James S. Blachly,^2,3^

Alice S. Mims,^2,3^ Christopher J. Walker,^3^ Michael C. Walker,^3,4^ Christopher C. Oakes,^2,3^

Shelley Orwick,^2,3^ Andrew J. Carroll,^15^ William G. Blum,^16^ Bayard L. Powell,^17^

Jonathan E. Kolitz,^18^ Joseph O. Moore,^19^ Robert J. Mayer,^20^ Richard A. Larson,^21^ Richard M. Stone,^22^ John C. Byrd,^23^ Klaus H. Metzeler,^12,13,24^ Tobias Herold,^1,12,13#^

and Ann-Kathrin Eisfeld,^2,3,#^

**PATIENTS AND METHODS**

**Treatment protocols**

***AMLCG-1999 trial***

**Induction therapy.** In the AMLCG-1999 trial^1^ (clinicaltrials.gov identifier NCT00266136; recruitment period, 1999 to 2011; patients included in this analysis were recruited between 1999 and 2004), patients aged <60 years were randomized to receive double induction with either one cycle of TAD-9 followed by one cycle of HAM on day 21 (TAD-HAM), or two cycles of HAM 21 days apart (HAM-HAM; TAD-9: thioguanine 100 mg/m2 twice daily on days 3-9, cytarabine 100 mg/m²/d continuous infusion on days 1+2 and 100 mg/m² twice daily on days 3-8, and daunorubicin 60 mg/m² on days 3-5; HAM: cytarabine 3 g/m² twice daily on days 1-3 and mitoxantrone 10 mg/m² on days 3-5). From 2002, 10% of patients aged ≤60 years (n=14 patients in this analysis) were randomized to a common standard arm of the German AML intergroup and received 7+3 induction therapy (cytarabine 100 mg/m²/d continuous infusion on days 1-7 and daunorubicin 60 mg/m² on days 3-5).^2^

**Postremission treatment.** Patients aged <60 years underwent upfront randomization to undergo consolidation either with one cycle of TAD-9 followed by three years of monthly cytarabine-based maintenance chemotherapy, or a single TAD-9 consolidation course followed by autologous stem cell transplantation (autoSCT) and no maintenance. Per the study protocol, all patients aged <60 years with HLA-matched sibling donors were to be offered allogeneic stem cell transplantation (alloSCT) in first complete remission (CR), irrespective of cytogenetic or molecular risk factors. Patients ≥60 years were randomized to receive induction therapy with one cycle of either TAD-9 or HAM, followed by a second HAM cycle on day 21 only if ≥5% residual blasts were present in the BM on day 16. All patients ≥60 years were to receive one cycle of TAD-9 consolidation followed by maintenance therapy.

***AMLCG-2008 trial***

In the AMLCG-2008 trial^3^ (NCT01382147; recruitment period, 2009 to 2012), patients <60 years and fit patients up to the age of 70 (‘younger’ patients) were randomized to receive either double induction chemotherapy with TAD-9 and HAM (21 days apart), or dose-dense induction therapy according to the sHAM regimen (cytarabine 3 g/m² [1 g/m² in patients ≥60 years] twice daily on days 1,2,8 and 9; and mitoxantrone 10 mg/m² on days 3,4,10 and 11). AlloSCT from an HLA-matched related or unrelated donor was the recommended postremission therapy for all younger patients achieving CR except those with favorable genetic features [defined as favorable cytogenetics or cytogenetically normal patients with mutated *NPM1* and no *FLT3* internal tandem duplications (*FLT3*-ITD) and good response to induction chemotherapy [<10% blasts in a bone marrow (BM) aspirate obtained on d16 after start of induction therapy]. For younger patients without a donor, those unable or unwilling to undergo alloSCT, and those with a favorable risk profile, postremission therapy consisted of one cycle of TAD-9 for consolidation, followed by 3 years of cytarabine-based maintenance therapy.

Less fit patients aged ≥60 years, and all patients aged ≥70 years, were randomized to receive induction therapy according to the HAM regimen (cytarabine, 1g/m² per dose) followed by a second HAM induction cycle on day 21 only if a BM aspirate on day 16 showed ≥5% blasts, or to dose-dense induction with sHAM (cytarabine, 1g/m² per dose). Postremission therapy in this group consisted of one cycle of TAD-9 for consolidation, followed by 3 years of maintenance therapy.

***CALGB/Alliance trials***

Patients in this study received intensive cytarabine/daunorubicin-based therapy on one of the following Cancer and Leukemia Group B (CALGB) frontline treatment protocols: 19808 (n=346), 10503 (n=322), 9720 (n=237), 9621 (n=180), 10201 (n=179), 8525 (n=99), 9222 (n=107), 10603 (n=78), 11002 (n=52), 10502 (n=39), 8923 (n=31), 9420 (n=23), 11001 (n=18), 9022 (n=16), 8821 (n=9), 8631 (n=2), 9120 (n=2), 8621 (n=1), 8721 (n=1), 10801 (n=1). Patients enrolled on CALGB 19808 were randomly assigned to receive induction chemotherapy with cytarabine, daunorubicin, and etoposide with or without PSC-833 (valspodar), a multidrug resistance protein inhibitor.^4^ On achievement of CR, patients were assigned to intensification with high-dose cytarabine (HiDAC) and etoposide for stem-cell mobilization followed by myeloablative treatment with busulfan and etoposide supported by autoSCT. Patients enrolled on CALGB 10503 were assigned to receive induction chemotherapy consisting of cytarabine, daunorubicin, and etoposide. Upon achievement of CR, patients received HiDAC and etoposide for stem-cell mobilization followed by myeloablative treatment with busulfan and etoposide supported by autoSCT. Patients not eligible for autoSCT received HiDAC. After intensification, patients received the DNA methyltransferase inhibitor decitabine for maintenance.^5^ In the CALGB 9720 study, patients aged 60 years and older, who were in their first remission after induction and consolidation chemotherapy were randomly assigned to no further therapy or a 90-day regimen of low-dose recombinant Interleukin-2 followed by 3-day of higher doses (n=49).^6^ Patients enrolled on CALGB 9621 were treated similarly to those on CALGB 19808, as previously reported.^7^ Patients enrolled on CALGB 10201 received standard cytarabine/daunorubicin induction chemotherapy, with or without the *BCL2*-targeting antisense oligonucleotide oblimersen sodium.^8^ Patients on CALGB 8525 were treated with induction chemotherapy consisting of cytarabine in combination with daunorubicin and were randomly assigned to consolidation with different doses of cytarabine followed by maintenance treatment.^9^ Patients on protocol CALGB 9222 received induction chemotherapy consisting of cytarabine in combination with daunorubicin followed by consolidation with one cycle of HiDAC. Different doses of mitoxantrone were explored, and the consolidation treatment was randomized to three cycles of monotherapy with HiDAC or consolidation with one cycle of HiDAC, a cycle of cyclophosphamide and etoposide, and one cycle of mitoxantrone and diaziquone.^10^ In CALGB 10603, cytarabine and daunorubicin followed by consolidation with HiDAC was applied with or without PKC-412.^11^ Patients on the CALGB 11002 trial received standard induction and post-remission therapy in combination with decitabine, with or without addition of bortezomib.^12^ For patients on CALGB 10502, bortezomib was added to both induction consisting of cytarabine and daunorubicin and to consolidation with two cycles of intermediate-dose cytarabine.^13^ Patients on CALGB 8923 received standard induction chemotherapy and those who achieved CR were randomly assigned to receive consolidation therapy with cytarabine alone or in combination with mitoxantrone.^14^ Patients on CALGB 9420 (n=9) received standard induction chemotherapy with cytarabine and daunorubicin, with or without the multidrug resistance protein inhibitor PSC-833.^15^ For the patients treated on the CALGB 11001 sorafenib was added to standard induction and HiDAC consolidation treatment, followed by sorafenib maintenance.^16^ Patients enrolled onto CALGB 9022 received induction chemotherapy consisting of cytarabine in combination with daunorubicin followed by consolidation with one cycle of HiDAC, a cycle of cyclophosphamide and etoposide, and one cycle of mitoxantrone and diaziquone.^17^ After induction consisting of cytarabine in combination with daunorubicin, the patients enrolled on CALGB 8821 received intensive post remission therapy with cytoxan/etoposide and diazaquone/mitoxantrone.^18^ Patients enrolled on CALGB 8621 received HiDAC for seven days in combination with mitoxantrone for the first three days. Patients enrolled on CALGB 8721 were randomly assigned to one of two remission induction regimens. Regimen I consisted of HiDAC plus asparaginase and regimen II consisted of HiDAC alone. Patients received two courses of treatment, beginning on days 1 and 8. A third course was begun on day 15 if patients had persistent blasts, that is, between 5% and 25% blasts in the day 14 marrow.

**Definition of clinical end points**

***AMLCG-1999 and AMLCG-2008 trials***

Clinical endpoints were defined, in accordance with generally accepted criteria,^19,20^ as follows: CR required a BM aspirate with cellularity greater than 20% and maturation of all cell lines, less than 5% blasts and no Auer rods; and in the peripheral blood, an absolute neutrophil count of ≥1.5 x 10^9^/L, platelet count of ≥100 x 10^9^/L, and no leukemic blasts; and no evidence of extramedullary leukemia. Relapse was defined by the presence of ≥5% BM blasts, or circulating leukemic blasts, or the development of extramedullary leukemia. Relapse-free survival (RFS) was measured from the date of CR until the date of relapse or death; patients alive and in CR were censored at last follow-up. Overall survival (OS) was measured from the date of study entry until the date of death, and patients alive at last follow-up were censored.

***CALGB/Alliance trials***

CR required an absolute neutrophil count ≥1.5 x 10^9^/L, with the exception for protocols CALGB 10503 and 10603, which required an absolute neutrophil count of ≥1.0 x 10^9^/L, platelet count ≥100 x 10^9^/L, no leukemic blasts in the blood, BM cellularity >20% with maturation of all cell lines, no Auer rods, ˂5% BM blast cells, and no evidence of extramedullary leukemia, all of which had persisted for at least one month. Relapse was defined by ≥5% BM blasts, circulating leukemic blasts, or the development of extramedullary leukemia. Disease-free survival (DFS) was measured from the date of CR until the date of relapse or death; patients alive and relapse-free at last follow-up were censored. OS was measured from the date on study until the date of death, and patients alive at last follow-up were censored.^21^

**Supplementary Materials and Methods**

Cytogenetic analyses of pretreatment BM and/or blood samples subjected to short-term (24- or 48-h) unstimulated cultures were performed by CALGB/Alliance- and AMLCG approved institutional laboratories, and the results were confirmed by karyotype review. For molecular analyses, mononuclear cells were enriched from pretreatment BM or PB by Ficoll density gradient centrifugation. Genomic DNA was extracted using the DNeasy Blood and Tissue Kit (QIAGEN, Hilden, Germany).

The mutational status of 80 protein coding genes *(AKT1, ARAF, ASXL1, ATM, AXL, BCL2, BCOR, BCORL1, BRAF, BRD4, BRINP3, BTK, CBL, CCND1, CCND2, CSNK1A1, CTNNB1, DNMT3A, ETV6, EZH2, FBXW7, FLT3* [for *FLT3* tyrosine kinase domain mutations *(FLT3*-TKD)]*, GATA1, GATA2, GSK3B, HIST1H1E, HNRNPK, IDH1, IDH2, IKZF1, IKZF3, ILR7, JAK1, JAK2, JAK3, KIT, KLHL6, KMT2A, KRAS, MAPK1, MAPK3, MED12, MYD88, NF1, NOTCH1, NPM1, NRAS, PHF6, PIK3CD, PIK3CG, PLCG2, PLEKHG5, PRKCB, PRKD3, PTEN, PTPN11, RAD21, RAF1, RUNX1, SAMHD1, SETBP1, SF1, SF3A1, SF3B1, SMARCA2, SMC1A, SMC3, SRSF2, STAG2, SYK, TET2, TGM7, TP53, TYK2, U2AF1, U2AF2, WT1, XPO1, ZMYM3, ZRSR2)* was determined by targeted amplicon sequencing using the MiSeq platform (Illumina, San Diego, CA). DNA library preparations were performed according to the manufacturer’s instructions. Briefly, samples were pooled and run on the MiSeq machine using the Illumina MiSeq Reagent Kit v3. Sequenced reads were aligned to the hg19 genome build using the Illumina Isis Banded Smith-Waterman aligner. Single nucleotide variant and indel calling were performed using MuTect and VarScan, respectively.^22,23^ The MuCor algorithm was used as the baseline for integrative mutation assessment.^24^ We only considered non-synonymous variants not listed in either the 1000 Genome database or dbSNP142-common variants as mutations. All called variants underwent visual inspection of the aligned reads using the Integrative Genomics Viewer (Broad Institute).^25^ All variants that occurred with variant allele fractions of <0.10 were considered wild-type; all variants that were sequenced to a depth of <15 reads were excluded from the analysis. In addition, variants were excluded when they occurred only in 1 read direction if sequenced in both directions, if the region contained many variants with low quality scores, or if they occurred in all analyzed samples including run controls. In addition, samples with high background noise were entirely excluded from analysis. Samples were considered non-evaluable for a specific gene if ≥85% of the amplicons covering the target regions within the coding sequence of the gene were sequenced to a depth of <15 reads.

The presence or absence of *FLT3*-ITD was determined as previously described,^26,27^ and testing for *CEBPA* gene mutations was performed using mutational profiling via targeted amplicon sequencing and/or transcriptional profiling.^28^ *FLT3-*ITD-to-wild-type allelic ratio was determined by PCR and fragment analysis from gDNA.^29^ Mutations in 68 genes recurrently mutated in myeloid malignancies, including *NPM1, FLT3, CEBPA, ASXL1, RUNX1*, and *TP53,* were identified by targeted gDNA sequencing, with a limit of detection of 2% variant allele frequency.^30,31^

**SUPPLEMENTARY REFERENCES**

1. Büchner T, Berdel WE, Haferlach C, Haferlach T, Schnittger S, Müller-Tidow C, et al. Age-related risk profile and chemotherapy dose response in acute myeloid leukemia: a study by the German Acute Myeloid Leukemia Cooperative Group. J Clin Oncol 2009;27:61-9.
2. Büchner T, Schlenk RF, Schaich M, Döhner K, Krahl R, Krauter J, et al. Acute Myeloid Leukemia (AML): different treatment strategies versus a common standard arm- combined prospective analysis by the German AML Intergroup. J Clin Oncol 2012;30:3604-10.
3. Braess J, Amler S, Kreuzer K-A, Spiekermann K, Lindemann HW, Lengfelder E, et al. Sequential high-dose cytarabine and mitoxantrone (S-HAM) versus standard double induction in acute myeloid leukemia-a phase 3 study. Leukemia 2018;32:2558-71.
4. Kolitz JE, George SL, Marcucci G, Vij R, Powell BL, Allen SL, et al. P-glycoprotein inhibition using valspodar (PSC-833) does not improve outcomes for patients under age 60 years with newly diagnosed acute myeloid leukemia: Cancer and Leukemia Group B study 19808. Blood. 2010;116:1413-21.
5. Blum W, Sanford BL, Klisovic R, DeAngelo DJ, Uy G, Powell BL, et al. Maintenance therapy with decitabine in younger adults with acute myeloid leukemia in first remission: a phase 2 Cancer and Leukemia Group B study (CALGB 10503). Leukemia. 2017;31:34-9.
6. Baer MR, George SL, Caligiuri MA, Sanford BL, Bothun SM, Mrózek K, et al. Low-dose interleukin-2 immunotherapy does not improve outcome of patients age 60 years and older with acute myeloid leukemia in first complete remission: Cancer and Leukemia Group B study 9720. J Clin Oncol 2008;26 4934-9.
7. Kolitz JE, George SL, Dodge RK, Hurd DD, Powell BL, Allen SL, et al. Dose escalation studies of cytarabine, daunorubicin, and etoposide with and without multidrug resistance modulation with PSC-833 in untreated adults with acute myeloid leukemia younger than 60 years: final induction results of Cancer and Leukemia Group B study 9621. J Clin Oncol 2004;22:4290-301.
8. Walker AR, Marcucci G, Yin J, Blum W, Stock W, Kohlschmidt J, et al. Phase 3 randomized trial of chemotherapy with or without oblimersen in older AML patients: CALGB 10201 (Alliance). Blood Adv 2021;5:2775-87.
9. Mayer RJ, Davis RB, Schiffer CA, Berg DT, Powell BL, Schulman P, et al. Intensive postremission chemotherapy in adults with acute myeloid leukemia. N Engl J Med. 1994;331:896-903.
10. Moore JO, George SL, Dodge RK, Amrein PC, Powell BL, Kolitz JE, et al. Sequential multiagent chemotherapy is not superior to high-dose cytarabine alone as postremission intensification therapy for acute myeloid leukemia in adults under 60 years of age: Cancer and Leukemia Group B study 9222. Blood. 2005;105:3420-7.
11. Stone RM, Mandrekar SJ, Sanford BL, Laumann K, Geyer S, Bloomfield CD, et al. Midostaurin plus chemotherapy for acute myeloid leukemia with a *FLT3* mutation. N Engl J Med 2017;377:454-64.
12. Roboz GJ, Mandrekar SJ, Desai P, Laumann K, Walker AR, Wang ES, et al. A randomized trial of 10 days of decitabine alone or with bortezomib in previously untreated older patients with acute myeloid leukemia: CALGB 11002 (Alliance). Blood Adv 2018;2:3608-17.
13. Attar EC, Johnson JL, Amrein PC, Lozanski G, Wadleigh M, DeAngelo DJ, et al. Bortezomib added to daunorubicin and cytarabine during induction therapy and to intermediate-dose cytarabine for consolidation in patients with previously untreated acute myeloid leukemia age 60 to 75 years: CALGB (Alliance) study 10502. J Clin Oncol 2013;31:923-9.
14. Stone RM, Berg DT, George SL, Dodge RK, Paciucci PA, Schulman P, et al. Granulocyte-macrophage colony-stimulating factor after initial chemotherapy for elderly patients with primary acute myelogenous leukemia. N Engl J Med 1995;332:1671-7.
15. Lee EJ, George SL, Caligiuri M Szatrowski TP, Powell BL, Lemke S, et al. Parallel phase I studies of daunorubicin given with cytarabine and etoposide with or without the multidrug resistance modulator PSC-833 in previously untreated patients 60 years of age or older with acute myeloid leukemia: Results of Cancer and Leukemia Group B study 9420. J Clin Oncol 1999;17:2831-9.
16. Uy GL, Mandrekar SJ, Laumann K, Marcucci G, Zhao W, Levis MJ, et al: A phase 2 study incorporating sorafenib into the chemotherapy for older adults with *FLT3*-mutated acute myeloid leukemia: CALGB 11001. Blood Adv 2017;1:331-40.
17. Moore JO, Dodge RK, Amrein PC, Kolitz J, Lee EJ, Powell B, et al. Granulocyte-colony stimulating factor (filgrastim) accelerates granulocyte recovery after intensive postremission chemotherapy for acute myeloid leukemia with aziridinyl benzoquinone and mitoxantrone: Cancer and Leukemia Group B study 9022. Blood. 1997;89:780-8.
18. Schiffer CA, Davis RB, Schulman P, Cooper B, Coyle T, Lee E, et al. Intensive post remission therapy of acute myeloid leukemia (AML) with cytoxan/etoposide (CY/VP16) and diazaquone/mitoxantrone (AZQ/MITO). Blood. 1991;78(suppl):460 (abstract 1829).
19. Cheson BD, Bennett JM, Kopecky KJ, Büchner T, Willman CL, Estey EH, et al. Revised recommendations of the International Working Group for diagnosis, standardization of response criteria, treatment outcomes, and reporting standards for therapeutic trials in acute myeloid leukemia. J Clin Oncol 2003;21:4642-9.
20. Döhner H, Estey E, Grimwade D, Amadori S, Appelbaum FR, Büchner T, et al. Diagnosis and management of AML in adults: 2017 ELN recommendations from an international expert panel. Blood 2017;129:424-47.
21. Cheson BD, Cassileth PA, Head DR, Schiffer CA, Bennett JM, Bloomfield CD, et al. Report of the National Cancer Institute-sponsored workshop on definitions of diagnosis and response in acute myeloid leukemia. J Clin Oncol 1990;8:813-9.
22. Cibulskis K, Lawrence MS, Carter SL, Sivachenko A, Jaffe D, Sougnez C, et al. Sensitive detection of somatic point mutations in impure and heterogeneous cancer samples. Nat Biotechnol 2013;31:213-9.
23. DePristo MA, Banks E, Poplin R, Garimella KV, Maguire JR, Hartl C, et al. A framework for variation discovery and genotyping using next-generation DNA sequencing data. Nat Genet 2011;43:491-8.
24. Kroll KW, Eisfeld A-K, Lozanski G, Bloomfield CD, Byrd JC, Blachly JS. MuCor: mutation aggregation and correlation. Bioinformatics 2016;32:1557-8.
25. Robinson JT, Thorvaldsdóttir H, Winckler W, Guttman M, Lander ES, Getz G, et al. Integrative genomics viewer. Nat Biotechnol 2011;29:24-6.
26. Whitman SP, Archer KJ, Feng L, Baldus C, Becknell B, Carlson BD, et al. Absence of the wild-type allele predicts poor prognosis in adult *de novo* acute myeloid leukemia with normal cytogenetics and the internal tandem duplication of *FLT3*: a Cancer and Leukemia Group B study. Cancer Res 2001;61:7233-9.
27. Schneider F, Hoster E, Unterhalt M, Schneider S, Dufour A, Benthaus T, et al. NPM1 but not *FLT3-ITD* mutations predict early blast cell clearance and CR rate in patients with normal karyotype AML (NK-AML) or high-risk myelodysplastic syndrome (MDS). Blood 2009;113:5250-3.
28. Taube F, Georgi JA, Kramer M, Stasik S, Middeke JM, Röllig C, et al. *CEBPA* mutations in 4708 patients with acute myeloid leukemia: differential impact of bZIP and TAD mutations on outcome. Blood 2022;139:87-103.
29. Thiede C, Steudel C, Mohr B, Schaich M, Schäkel U, Platzbecker U, et al. Analysis of FLT3-activating mutations in 979 patients with acute myelogenous leukemia: association with FAB subtypes and identification of subgroups with poor prognosis. Blood 2002;99:4326-35.
30. Metzeler K, Herold T, Rothenberg-Thurley M, Amler S, Sauerland MC, Görlich D, et al. Spectrum and prognostic relevance of driver gene mutations in acute myeloid leukemia. Blood 2016;128:686-98.
31. Rothenberg-Thurley M, Amler S, Goerlich D, Köhnke T, Konstandin NP, Schneider S, et al. Persistence of pre-leukemic clones during first remission and risk of relapse in acute myeloid leukemia. Leukemia 2018;32:1598-608.
